# Synthesis and Preclinical Characterization of [^18^F]FPBZA: A Novel PET Probe for Melanoma

**DOI:** 10.1155/2014/912498

**Published:** 2014-09-01

**Authors:** Shih-Yen Wu, Shih-Pin Huang, Yen-Chen Lo, Ren-Shyan Liu, Shyh-Jen Wang, Wuu-Jyh Lin, Chih-Chieh Shen, Hsin-Ell Wang

**Affiliations:** ^1^Department of Biomedical Imaging and Radiological Sciences, National Yang-Ming University, No. 155 Li-Nong Street, Section 2, Pei-Tou, Taipei 11221, Taiwan; ^2^Department of Nuclear Medicine, Faculty of Medicine, National Yang-Ming University, No. 155 Li-Nong Street, Section 2, Pei-Tou, Taipei 11221, Taiwan; ^3^Institute of Nuclear Energy Research, Atomic Energy Council, No. 1000 Wenhua Road, Jiaan Village, Longtan, Taoyuan County 32546, Taiwan; ^4^Department of Nuclear Medicine, Cheng Hsin General Hospital, No. 45 Cheng Hsin Street, Pei-Tou, Taipei 11220, Taiwan

## Abstract

*Introduction*. Benzamide can specifically bind to melanoma cells. A ^18^F-labeled benzamide derivative, [^18^F]*N*-(2-diethylaminoethyl)-4-[2-(2-(2-fluoroethoxy) ethoxy)ethoxy]benzamide ([^18^F]FPBZA), was developed as a promising PET probe for primary and metastatic melanoma. *Methods*. [^18^F]FPBZA was synthesized via a one-step radiofluorination in this study. The specific uptake of [^18^F]FPBZA was studied in B16F0 melanoma cells, A375 amelanotic melanoma cells, and NB-DNJ-pretreated B16F0 melanoma cells. The biological characterization of [^18^F]FPBZA was performed on mice bearing B16F0 melanoma, A375 amelanotic melanoma, or inflammation lesion. *Results*. [^18^F]FPBZA can be prepared efficiently with a yield of 40–50%. The uptake of [^18^F]FPBZA by B16F0 melanoma cells was significantly higher than those by A375 tumor cells and NB-DNJ-pretreated B16F0 melanoma cells. B16F0 melanoma displayed prominent uptake of [^18^F]FPBZA at 2 h (7.81 ± 0.82 %ID/g), compared with A375 tumor and inflammation lesion (3.00 ± 0.71 and 1.67 ± 0.56 %ID/g, resp.). [^18^F]FPBZA microPET scan clearly delineated B16F0 melanoma but not A375 tumor and inflammation lesion. In mice bearing pulmonary metastases, the lung radioactivity reached 4.77 ± 0.36 %ID/g at 2 h (versus 1.16 ± 0.23 %ID/g in normal mice). *Conclusions*. Our results suggested that [^18^F]FPBZA PET would provide a promising and specific approach for the detection of primary and metastatic melanoma lesions.

## 1. Introduction

Melanoma, with a steadily increasing incidence rate for the past 30 years, has become a serious public health problem worldwide [1, 2]. In the United States, the incidence rose from 7.5 cases per 100,000 inhabitants in 1973 to 21.9 cases per 100,000 inhabitants in 2002 [3].

Malignant melanoma is very aggressive and more likely than other skin cancers to spread to other parts of the body, including brain, lungs, liver, and lymph nodes [4]. The prognosis and the outcome of melanoma treatment heavily depend on the clinical stage of the disease at the time of diagnosis. Melanoma is highly curable if detected in its earliest stages and treated properly [4–6]. Early stage melanoma can be cured by surgical excision and the 5-year relative survival rates for persons with melanoma are >90% [1, 5]. However, advanced melanoma with distant tumor spread is largely refractory to existing therapies and has a poor prognosis with 5-year survival rate of 15% [1, 5, 7, 8]. Thus, early diagnosis and accurate disease staging are required for optimizing the treatment plan and can avoid unnecessary and potentially harmful surgical therapies that would yield no improvement in survival.

Positron emission tomography (PET) imaging technique offers unique advantages including high sensitivity as well as deep tissue tomographic and repetitive imaging capabilities. Further, this imaging approach is directly translatable to clinical application. The most generally used radiotracer in clinical practice is [^18^F]fluorodeoxyglucose ([^18^F]FDG). [^18^F]FDG is actively taken at the cellular level, then phosphorylated, and no longer metabolized, and thus it remains trapped within the cells. [^18^F]FDG PET was recommended for staging and detecting recurrent melanoma [9, 10]. However, it was reported that the sensitivity of [^18^F]FDG PET was unacceptably low for the initial assessment of early-stage malignant melanoma [11, 12]. [^18^F]FDG PET scans also failed to detect the pulmonary and brain metastases and should be limited to staging patients with more advanced melanoma [13, 14]. Moreover, [^18^F]FDG displays relatively poor selectivity for distinguishing tumor from inflammatory tissue. Increased glucose metabolism in inflammatory tissues is often the main cause of false-positive [^18^F]FDG PET findings in oncology.

Primary melanoma most commonly arises from the skin; however, it can also develop in other areas, including the eye and mucosa. A key feature of melanoma is the extensive melanin present in most tumor cells, making it a very attractive target for both diagnosis and treatment. Various radiolabeled benzamide analogues have been developed for targeting melanin with high selectivity and affinity [15–18]. Among these derivatives,* N*-(2-diethylaminoethyl)-4-[^18^F]fluorobenzamide ([^18^F]FBZA) exhibited high tumor uptake and was considered as a promising candidate for clinical study [17, 18]. The synthesis of [^18^F]FBZA is conducted via conjugation of a ^18^F-synthon (*N*-succinimidyl-4-[^18^F]fluorobenzoate, [^18^F]SFB) to the primary amino group of* N,N*-diethylethylenediamine (DEDA). This method requires a multistep synthesis and is time-consuming and laborious. Furthermore, it is difficult to make the multistep radiosynthesis fully automatic, which in turn sets a high technical barrier for [^18^F]FBZA in the clinical use.

**Figure 1:**
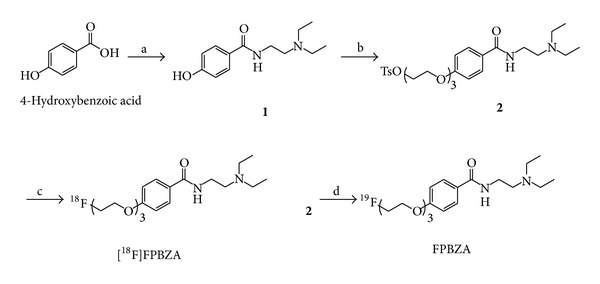
Synthesis of [^18^F]FPBZA and its authentic compounds. a: (i) thionyl chloride (ii) DEDA, K_2_CO_3_, THF; b: triethylene glycol di-*p*-tosylate, K_2_CO_3_, MeCN; c: K[^18^F]/K2.2.2, DMSO; d: TBAF, THF.

One of the commonly used methods to append a F-18 atom involves conjugating a fluoroalkyl group to the compound; however, the results were not always promising. The ^18^F-labeled tracers with an additional fluoroalkyl moiety have higher lipophilicity and tend to display a higher nonspecific binding to the normal organs. Polyethylene glycol (PEG) is a component that has been widely used as a modifier of drugs and has been approved by the food and drug administration of United States of America. This study developed an easy-to-synthesize method to prepare a [^18^F]fluoroPEGylated radiotracer for melanoma detection. We modified the phenol moiety of benzamide with a short chain PEG and then labeled with ^18^F to afford [^18^F]*N*-(2-diethylaminoethyl)-4-[2-(2-(2-fluoroethoxy)ethoxy)ethoxy]benzamide ([^18^F]FPBZA, Figure 1). This study presented a preclinical assessment of [^18^F]FPBZA as a PET probe for specific imaging of melanin. To achieve this purpose, biological characterizations of [^18^F]FPBZA were performed in C57BL/6 mice bearing subcutaneous or pulmonary metastatic B16F0 murine melanoma with high melanin expression, in BALB/c nude mice bearing A375 human amelanotic melanoma, and in C57BL/6 mice bearing turpentine-induced aseptic inflammation.

## 2. Materials and Methods

### 2.1. Reagents and Instruments

All reagents and solvents were purchased from commercial suppliers and were used without further purification. Triethylene glycol di-*p*-tosylate was synthesized following the procedures described in literature [19]. Thin layer chromatography (TLC) was performed on silica gel F_254_ aluminum-backed plates (Merck, Darmstadt, Germany) with visualization under UV (254 nm). NMR spectra were recorded on a NMR spectrometer (Bruker, Germany) operating at 400 MHz for ^1^H NMR spectra and 100 MHz for ^13^C NMR spectra at Instrumentation Resource Center of National Yang-Ming University. All chemical shift values were reported in ppm (*δ*). Electrospray ionization-mass spectra (ESI-MS) were acquired on a FINNIGAN LCQ mass spectrometer at Instrumentation Resource Center of National Taiwan University. Analytic as well as semipreparative high performance liquid chromatography (HPLC) was performed with a Waters 600E pump equipped with a Waters 2998 photodiode array detector and a flow count radio detector (Bioscan, Washington DC) for gamma ray detection. Radioactivity was assayed using a Capintec CRC-15R dose calibrator (Ramsey, NJ) or a *γ*-scintillation counter (Wallac 1470 Wizard automatic gamma counter, Perkin-Elmer, Waltham, MA).

### 2.2. Synthesis of [^18^F]FPBZA

#### 2.2.1. Synthesis of 4-Hydroxy-*N*-(2-diethylaminoethyl)benzamide (**1**)

A solution of 4-hydroxybenzoic acid (500 mg, 3.6 mmol) in thionyl chloride (5 mL) was refluxed for 5 h. Excess thionyl chloride was removed under reduced pressure. The residue was then redissolved in THF (10 mL). To this solution was added DEDA (462 mg, 4.0 mmol) and K_2_CO_3_ (1.0 g, 7.2 mmol). The reaction mixture was stirred at ambient temperature overnight, diluted with H_2_O and extracted with dichloromethane. The combined organic layers were washed with H_2_O, dried over MgSO_4_, and evaporated. Column chromatography of the crude product on silica gel eluting with MeOH/CH_2_Cl_2_ (1/10) afforded compound** 1** (790 mg, 3.34 mmol, 93% yield) as a yellowish solid. ^1^H NMR (400 MHz, acetone-d6) *δ* (ppm) 1.03 (t, 6H, *J* = 7.1 Hz), 2.58 (q, 4H, *J* = 7.1 Hz), 2.65 (t, 2H, *J* = 6.7 Hz), 3.44 (q, 2H, *J* = 6.3 Hz), and 6.89 (d, 2H, *J* = 8.7 Hz), 7.76 (d, 2H, *J* = 8.7 Hz). ^13^C NMR (100 MHz, acetone-d6) *δ* (ppm) 11.6, 37.6, 47.0, 52.0, 114.9, 126.3, 128.8, 160.1, 166.1. ESI-MS* m/z* calculated for C_13_H_20_N_2_O_2_: 236.15 found [M + H]^+^: 237.16.

#### 2.2.2. Synthesis of* N*-(2-Diethylaminoethyl)-4-[2-(2-(2-tosyloxyethoxy)ethoxy)-ethoxy]benzamide (**2**)

To a solution of** 1** (1.0 g, 4.23 mmol) in anhydrous acetonitrile (MeCN; 50 mL) were added K_2_CO_3_ (2.93 g, 21.18 mmol) and triethylene glycol di-*p*-tosylate (5.82 g, 12.71 mmol). The reaction mixture was heated to reflux for 4 h. The solvent was removed by rotary evaporator. The residue was redissolved with H_2_O and extracted with dichloromethane. The combined organic layers were dried over MgSO_4_ and concentrated. Column chromatography of the crude product on silica gel eluting with MeOH/CH_2_Cl_2_ (1/10) afforded the tosyl precursor (compound** 2**, 1.13 g, 2.16 mmol, 51% yield) as an yellowish oil. ^1^H NMR (400 MHz, CDCl_3_) shows *δ* (ppm) 1.08 (t, 6H, *J* = 7.2 Hz), 2.43 (s, 4H), 2.64 (q, 4H, *J* = 7.1 Hz), 2.72 (t, 2H, *J* = 5.9 Hz), 3.52 (q, 2H, *J* = 5.5 Hz), 3.60–3.71 (m, 6H), 3.83 (t, 2H, *J* = 4.8 Hz), 4.13–4.17 (m, 4H), 6.92 (d, 2H, *J* = 8.8 Hz), 7.33 (d, 2H, *J* = 8.2 Hz), and 7.80–7.75 (m, 4H). ^13^C NMR (100 MHz, CDCl_3_) shows *δ* (ppm) 11.6, 21.6, 37.1, 47.1, 51.6, 67.5, 68.8, 69.2, 69.7, 70.8, 114.3, 127.1, 128.0, 128.7, 129.8, 133.0, 144.8, 161.2, and 166.9. ESI-MS* m/z* calculated for C_26_H_38_N_2_O_7_S: 522.24 found [M + H]^+^: 523.25.

#### 2.2.3. Synthesis of* N*-(2-Diethylaminoethyl)-4-[2-(2-(2-fluoroethoxy)ethoxy)ethoxy]-benzamide (FPBZA)

To a solution of compound** 2** (104 mg, 0.2 mmol) in THF (4 mL) was added tetrabutylammonium fluoride (TBAF) solution in THF (1 M, 0.4 mL). The resulting mixture was refluxed for 12 h. The solvent was evaporated and the crude product was purified by column chromatography on silica gel eluting with MeOH/CH_2_Cl_2_ (1/10) afforded FPBZA (compound** 3**, 48 mg, 0.13 mmol, and 65% yield) as an yellowish oil. ^1^H NMR (400 MHz, CDCl_3_) shows *δ* (ppm) 1.07 (t, 6H, *J* = 7.1 Hz), 2.62 (q, 4H, *J* = 7.1 Hz), 2.70 (t, 2H, *J* = 5.9 Hz), 3.52 (q, 2H, *J* = 5.3 Hz), 3.70–3.80 (m, 6H), 3.88 (t, 2H, *J* = 4.8 Hz), 4.17 (t, 2H, *J* = 4.8 Hz), 4.49–4.63 (dd, 2H, *J*_HF_ = 47.6, *J*_HH_ = 4.2), 6.94 (d, 2H, *J* = 8.8 Hz), and 7.76 (d, 2H, *J* = 8.8 Hz). ^13^C NMR (100 MHz, CDCl_3_) shows *δ* (ppm) 11.6, 37.1, 46.9, 51.6, 67.5, 69.4, 70.4, 70.8, 70.9, 83.1, 114.3, 127.1, 128.7, 161.2, and 166.69. ESI-MS* m/z* calculated for C_19_H_31_FN_2_O_4_: 370.23 found [M + H]^+^: 371.23.

#### 2.2.4. Preparation of* N*-(2-Diethylaminoethyl)-4-[2-(2-(2-[^18^F]fluoroethoxy)ethoxy)-ethoxy]benzamide ([^18^F]FPBZA)

[^18^F]HF was produced by irradiation of 95% ^18^O-enriched water with a 17-MeV proton beam at 18 *μ*A. At the end of irradiation, [^18^F]HF was transferred to a vial by helium gas pressure and then transferred through a QMA Sep-Pak cartridge. The [^18^F]fluoride trapped on the cartridge was desorbed by elution with 0.8 mL of K_2_CO_3_/Kryptofix 2.2.2 (K2.2.2) solution (22 mg of K2.2.2 and 4 mg of K_2_CO_3_; in MeCN/H_2_O 4/1) into a reaction vessel. The K[^18^F]/K2.2.2 solution in the reaction vessel was heated at 100°C under reduced pressure. The azeotropic drying was repeated twice by further addition of anhydrous MeCN (2 × 0.8 mL). To the dry residue was added compound** 2** (2.0 mg) in anhydrous dimethyl sulfoxide (DMSO) and the resulting mixture was heated at 100°C for 5 min. After cooling to room temperature, the reaction mixture was diluted with HPLC mobile phase and filtered with a 0.45 *μ*m membrane filter (Millipore, Bedford, MA). The crude product was injected into a C18 semipreparative HPLC column (Merck Purospher STAR RP-18e, 10 × 250 mm). The mobile phase started from 95% solvent A (0.1% trifluoroacetic acid in water) and 5% solvent B (0.1% trifluoroacetic acid in MeCN) and then ramped to 35% solvent A and 65% solvent B at 20 min at a flow rate of 4 mL/min. The desired fractions were combined, evaporated under reduced pressure, redissolved in normal saline, and filtered through a 0.22 *μ*m aseptic membrane filter (Millipore, Bedford, MA) to afford the final product. Radiochemical purity of the final product was analyzed by analytic HPLC using a Phenomenex Luna C18 column (4 × 250 mm) with a mobile phase consisting of MeCN/10 mM ammonium formate buffer (21/79) at a flow rate of 1 mL/min.

### 2.3. Serum Stability

[^18^F]FPBZA (7.4 MBq) was incubated in 0.5 mL of human serum. At designated time points (30, 60, and 120 min), 75 *μ*L of the sample was taken and mixed with MeCN (250 *μ*L). The resulting mixture was vortexed intermittently for 1 minute, then followed by centrifugation at 1,000 g for 10 min to pellet the precipitated serum proteins. The radioactivity of the supernatant and the precipitate was measured with a *γ*-scintillation counter. The radioactive components in the supernatant were assayed on an analytic HPLC as described above.

### 2.4. Lipophilicity

[^18^F]FPBZA (74 kBq) was added to a mixture of 1-octanol (1 mL) and phosphate buffer saline (PBS; pH 7.4, 1 mL); then, the mixture was vigorously vortexed for 5 min. After subsequent centrifugation at 3,000 rpm for 5 min, aliquots (100 *μ*L) were taken from each phase and the radioactivity was determined with a *γ*-scintillation counter. The partition coefficient was expressed as log⁡ *P* = log⁡_10_⁡ (counts in 1-octanol/counts in PBS).

### 2.5. Cellular Uptake Study

B16F0 melanoma cells and A375 amelanotic melanoma cells were obtained from Bioresource Collection and Research Center (Taiwa,) and were cultured in Dulbecco's modified Eagle high-glucose medium (Gibco, Carlsbad, CA) containing 10% fetal bovine serum at 37°C in a humidified atmosphere of 5% CO_2_.

For cellular uptake assay, B16F0 cells and A375 cells were trypsinized and grown overnight in 6-well culture plates (5 × 10^5^ cells/1.0 mL/well), and the medium was changed before experiment. [^18^F]FPBZA (37 kBq/1.0 mL/well) was added to each well and incubated at 37°C for 5, 15, 30, 60, and 120 min. Triplicates were carried out for each time point. One milliliter of ice-cold PBS was used to intercept the uptake of tracer. The supernatants were aspirated and the cells rinsed twice with 1 mL of ice-cold PBS. Then, cells in each well were harvested with 0.5 mL of trypsin-EDTA and washed twice with 1 mL of ice-cold PBS. A 10 *μ*L of cell suspension was taken to access cell viability with trypan blue and to count the number of viable cells under the microscopy. The radioactivity of the cell suspensions was measured with a *γ*-scintillation counter and normalized to the number of viable cells. Cellular uptake of [^18^F]FPBZA was expressed as the percentage of the administered dose that had accumulated per million cells (%AD/10^6^ cells).

### 2.6. Effect of Melanin Content on Cellular Uptake of [^18^F]FPBZA

To further demonstrate the specific binding of [^18^F]FPBZA to cellular melanin, an in vitro study using B16F0 cells, with and without pretreatment of* N*-butyldeoxynojirimycin (NB-DNJ, a tyrosinase inhibitior that can retard melanin synthesis), was conducted [20]. Briefly, B16F0 cells were seeded into 6-well plates at a density of 2 × 10^5^ cells per well and incubated overnight at 37°C. After cells had adhered, the growth medium in each well was replaced with fresh medium containing NB-DNJ (0, 0.5 and 1 mM) and replenished every 24 h thereafter. After 48 h of treatment, cells were washed with PBS and [^18^F]FPBZA (37 kBq/1.0 mL/well) was added to each well and incubated at 37°C for 120 min. One milliliter of ice-cold PBS was used to intercept the uptake of tracer. The supernatants were removed and the cells were rinsed twice with 1 mL of ice-cold PBS. The cells in each well were harvested with 0.5 mL of trypsin-EDTA and washed twice with 1 mL of ice-cold PBS. The radioactivity of the cell suspensions was measured with a *γ*-scintillation counter and normalized to the number of viable cells. The cellular uptake of [^18^F]FPBZA was expressed as %AD/10^6^ cells.

### 2.7. Animal Model

The animal experiments were approved by the Institutional Animal Care and Use Committee of the National Yang-Ming University. For the subcutaneous tumor models, male C57BL/6 mice were injected in the right flank with 0.1 mL of B16F0 melanoma cell suspension of 5 × 10^6^ cells/mL, and BALB/c nude mice were inoculated in the right flank with 2 × 10^6^ A375 amelanotic melanoma cells. Biological studies were conducted when the tumor burden reached 50~150 mm^3^. For the pulmonary metastasis animal model, mice were injected via the tail vein with 2 × 10^5^ B16F0 melanoma cells in 0.2 mL of PBS as previously described [21]. Animal studies were performed 15 days following tumor cell inoculation intravenously. The inflammation was induced by intramuscular injection of 0.1 mL of turpentine into the right flank of male C57BL/6 mice at 4 days before the biological experiment was conducted [22].

### 2.8. Biodistribution Studies

Mice bearing tumor or inflammation lesion were injected with 3.7 MBq of [^18^F]FPBZA in 0.1 mL of normal saline through lateral tail veins. Five mice were sacrificed with CO_2_ at each designated time point following injection. Blood samples were obtained by cardiac puncture. Tumor, inflammation lesion, and major organs were excised, and parts of these tissues/organs were weighed and assayed for radioactivity with a *γ*-scintillation counter. The results were presented as percentage injected dose per gram of tissue (%ID/g). Values were expressed as mean ± SD for a group of five animals.

### 2.9. MicroPET and MicroPET/CT Scanning

MicroPET images were obtained using the R4 system (Concorde Microsystems, Knoxville, TN, USA), and this produced 63 image slices over a 7.89 cm axial field of view (FOV), with a slice thickness of about 1.25 mm. The in-plane spatial resolution at the center of the FOV was about 1.6 mm using the ordered-subsets expectation maximization (OSEM) reconstruction method. Each tumor-bearing mouse was anesthetized with 0.2% isoflurane (Abbott Laboratories, Queensborough, Kent, UK) using a vaporizer system (A.M. Bickford, Wales Center, NY) and injected with approximately 1.85–3.7 MBq of [^18^F]FPBZA in 0.1 mL of physiologic saline through the lateral tail vein. Animals were scanned for 10 min at each time point (30, 60, and 120 min after injection), with the long axis of the animal parallel to the long axis of the scanner. All images were reconstructed with the OSEM method, with a 128 × 128 pixel image matrix, 16 subsets, 4 iterations, and use of a Gaussian filter. For data quantitative analysis, a region of interest (ROI) was placed on each tumor, inflammatory lesion and normal organs. The average radioactivity was obtained from the average pixel value within the multiple ROI volume. AsiPro software (Concorde Microsystems) was used for viewing microPET images and for data analysis. The counts in each ROI were converted to radioactivity per gram of tissue (nCi/g), assuming a tissue density of 1 g/mL and were then normalized to percentage of injected dose per gram of tissue (%ID/g).

### 2.10. Statistical Analysis

Results were expressed as mean ± SD. Statistical analysis was performed using Student's *t*-test for unpaired data. A 95% confidence level was chosen to determine the significance of differences between groups, with a *P* value of less than 0.05 indicating a significant difference.

## 3. Results

### 3.1. Chemistry and Radiochemistry

The preparation of all the intermediates and final product can be achieved in a straightforward manner (Figure 1). Compound** 1** was prepared from 4-hydroxybenzoic acid by the treatment of thionyl chloride in THF, followed by addition of DEDA to provide >90% yield of the desired product. Compound** 2**, the tosyl precursor for radiofluorination, was prepared successfully by coupling the free phenolic hydroxyl groups of compound** 1** with triethylene glycol di-*p*-tosylate in 51% yield. Reaction of compound** 2** with TBAF in THF produced the authentic FPBZA in 65% yield. All compounds were fully characterized by ^1^H NMR, ^13^C NMR, and mass spectra.

**Figure 2:**
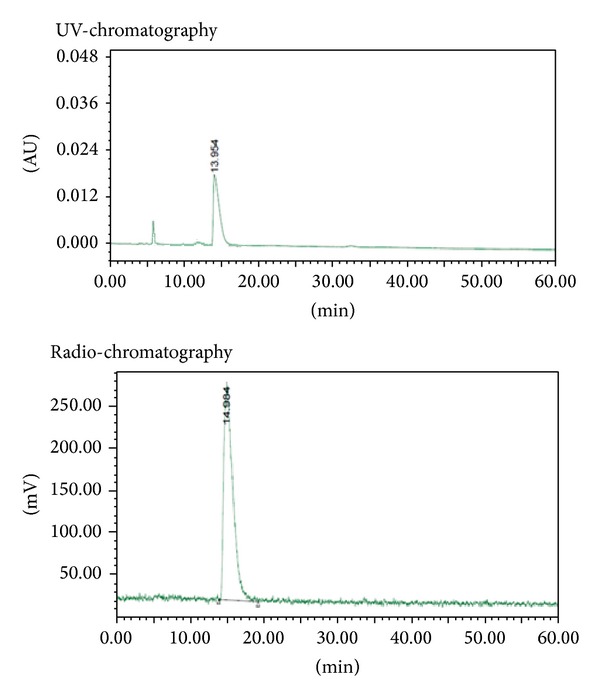
Analytic HPLC chromatogram of [^18^F]FPBZA with coinjection of the authentic compound FPBZA.

The radiofluorination of compound** 2** with K[^18^F]F/K2.2.2 in dry DMSO at 100°C for 5 min produced [^18^F]FPBZA with an average radiolabeling efficiency of >90% (*n* = 5). After purification by semipreparative HPLC, [^18^F]FPBZA final product was obtained with a radiochemical yield of 40–50%, a radiochemical purity of >97%, and a specific activity of 30–40 GBq/*μ*mol. The synthesis time was ~40 min from the end of bombardment, including the process of purification and formulation. The identity of [^18^F]FPBZA was confirmed by coinjection with the authentic FPBZA (Figure 2).

### 3.2. Characterization of [^18^F]FPBZA

After treating the [^18^F]FPBZA-added serum with acetonitrile followed by centrifugation, only a small portion of radioactivity was found in the serum protein precipitate and >90% of the radioactive components retained in solution. Intact [^18^F]FPBZA accounted for >95% of the radioactivity in solution within 2 h of incubation (see Figure  S1 in the Supplementary Material available online at http://dx.doi.org/10.1155/2014/912498). Defluorination of [^18^F]FPBZA in human serum was not observed. [^18^F]FPBZA, with a partition coefficient (log *P*) of −0.48, is more hydrophilic than the unPEGylated benzamide derivative [^18^F]FBZA (log *P* = 1.7) [17].

### 3.3. Cellular Uptake of [^18^F]FPBZA

**Figure 3:**
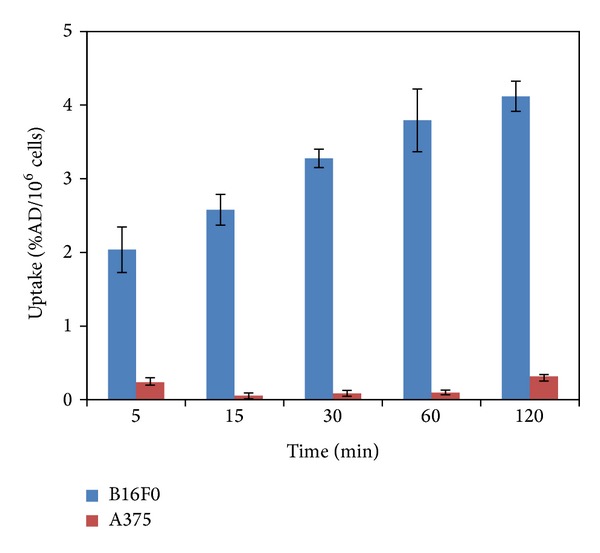
In vitro uptake studies of [^18^F]FPBZA in B16F0 melanoma cells and A375 amelanotic melanoma cells over time (*n* = 3; mean ± SD).

Cellular uptake study of [^18^F]FPBZA in B16F0 melanoma cells and A375 amelanotic melanoma cells was conducted to assess the specific binding of [^18^F]FPBZA toward high melanin-expressing tumor cells. Rapid and significant association of [^18^F]FPBZA with B16F0 cells was observed early after 5 min of incubation (2.58 ± 0.21 %AD/10^6^ cells) (Figure 3). Continued exposure led to an appreciably increased cellular uptake up to 2 h. Accumulation of [^18^F]FPBZA in B16F0 melanoma cells was significantly higher than that in amelanotic A375 cells (4.12 ± 0.20 %AD/10^6^ cells and 0.31 ± 0.06 %AD/10^6^ cells, respectively, after 2 h of incubation; *P* < 0.05). The uptake of [^18^F]FPBZA in B16F0 melanoma cells presented a time-dependent increase, while that in A375 cells did not.

**Figure 4:**
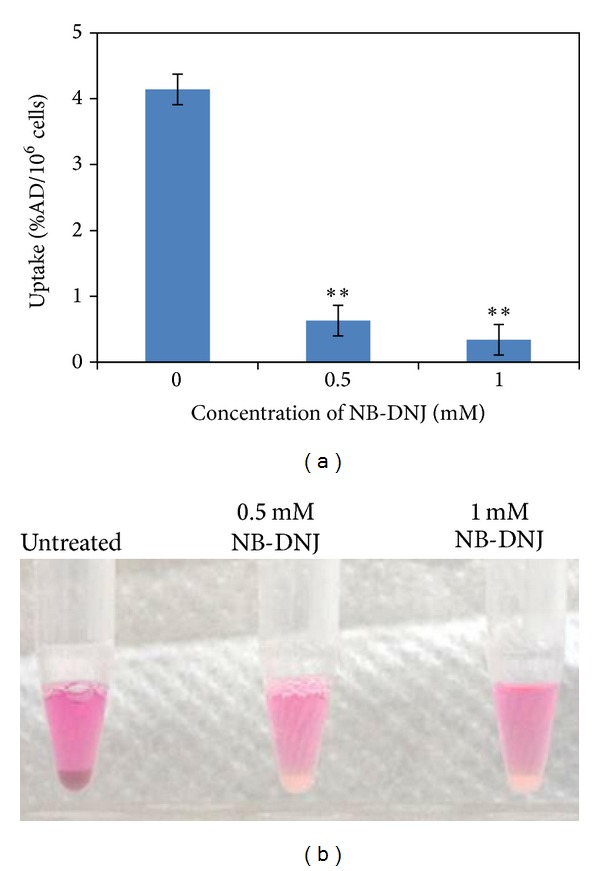
(a) Inhibition of [^18^F]FPBZA uptake in B16F0 cells by the pretreatment with various concentration of NB-DNJ for 48 h. The results were expressed in percentage of administered radioactivity per million cells and presented as mean ± SD (*n* = 3). **P < 0.01, compared with the corresponding untreated group. (b) Photos of B16F0 cell pellets with and without NB-DNJ pretreatment (untreated cells were used as control).

After pretreating B16F0 melanoma cells with 0.5 mM and 1.0 mM of NB-DNJ for 2 days, the cellular uptake of [^18^F]FPBZA dramatically decreased from 4.12 ± 0.20 %AD/10^6^ cells to 0.63 ± 0.22 and 0.34 ± 0.23 %AD/10^6^ cells, respectively, after 2 h of incubation (*P* < 0.01 in both conditions; Figure 4(a)). The NB-DNJ-pretreated B16F0 cells contained less melanin and showed a significantly reduced pigmentation compared with that of the control (Figure 4(b)).

### 3.4. Biodistribution Studies

Table 1 summarized the results of biodistribution studies after intravenous administration of [^18^F]FPBZA into mice bearing various tumor or inflammation lesion. In B16F0 melanoma-bearing C57BL/6 mice, rapid and high tumor uptake (10.37 ± 1.13 %ID/g) was observed at 0.5 h postinjection (p.i.), then declined slowly to 8.31 ± 1.00 and 7.81 ± 0.82 %ID/g at 1 and 2 h p.i., respectively. Most normal tissues displayed relatively low radioactivity accumulation through the whole study period, compared with that of tumor. The radioactivity remaining in muscle and blood was 2.83 ± 0.46 and 3.78 ± 0.41 %ID/g at 0.5 h p.i., respectively, and reduced to 1.16 ± 0.09 and 2.17 ± 0.10 %ID/g at 2 h p.i., respectively. A prolonged [^18^F]FPBZA retention in tumor, compared with those in normal tissues, rendered favorable tumor-to-normal tissues ratios for tumor detection. The tumor-to-muscle ratio was 3.72 at 0.5 h p.i., and significantly increased to 6.60 at 2 h p.i.. The tumor-to-liver and tumor-to-blood ratios also reached 4.49 and 3.52 at 2 h p.i., respectively. The bony uptake increased slightly over time and reached 3.21 ± 0.16 %ID/g at 2 h p.i.. The accumulation of [^18^F]FPBZA in inflammation lesion was low (1.67 ± 0.56 %ID/g) and was only slightly higher than that in muscle (1.28 ± 0.07 %ID/g; *P* > 0.1).

In A375 amelanotic melanoma-bearing nude mice, the tumor uptake was low (3.00 ± 0.71 %ID/g) and the tumor-to-muscle ratio was just 2.72 at 2 h p.i.. Although high uptake of [^18^F]FPBZA was observed in the pigmented eyes of C57BL/6 mice (7.69 ± 1.56 %ID/g), there was a minor uptake in the unpigmented eyes of nude mice (2.91 ± 0.45 %ID/g).

### 3.5. MicroPET and MicroPET/CT Imaging

**Figure 5:**
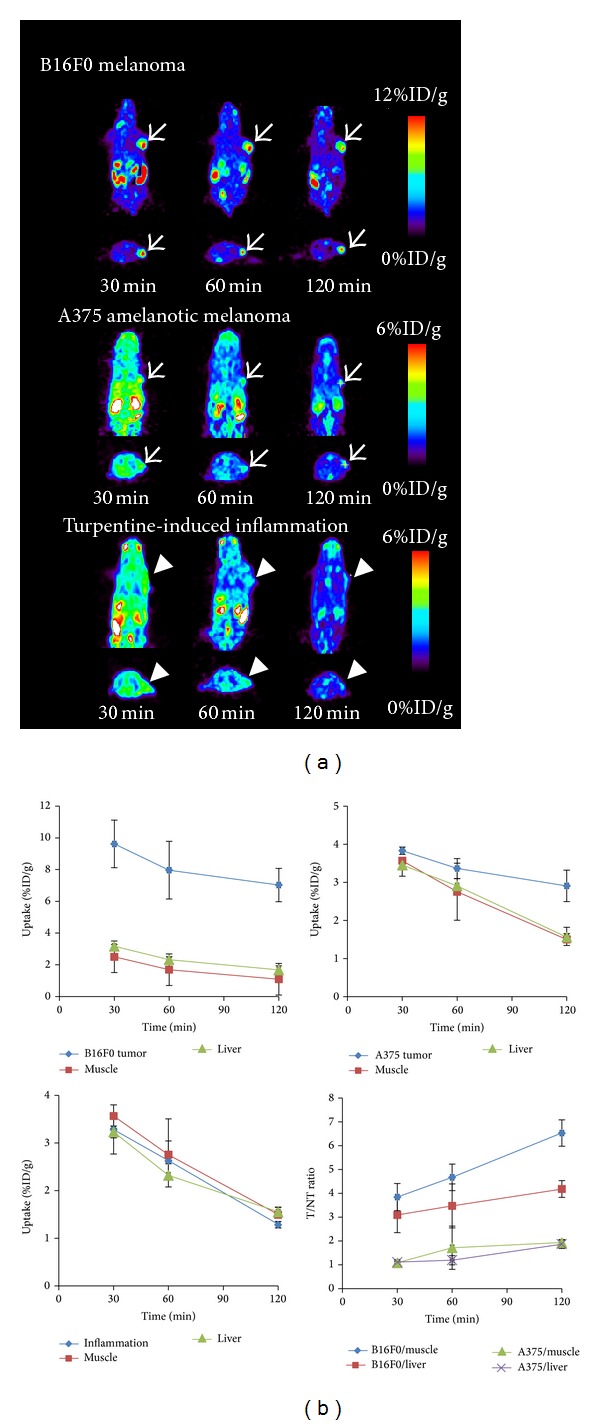
(a) Representative coronal (top) and transaxial (bottom) [^18^F]FPBZA microPET images of mice bearing B16F0 tumor, A375 tumor, and turpentine-induced inflammation. Static microPET images were obtained at 0.5, 1, and 2 h after bolus injection of 1.85–3.7 MBq of [^18^F]FPBZA via the tail vein. Arrows indicated tumors and arrow head indicated inflammation regions. (b) Time-activity curves of tumors, inflammation lesion, and normal tissues in various mouse models derived from microPET images after intravenous injection of [^18^F]FPBZA. SUVs were expressed in  %ID/g ± SD (*n* = 3).

[^18^F]FPBZA microPET imaging of B16F0 melanoma-bearing C57BL/6 mice showed excellent tumor-to-background contrast (Figure 5(a)). Tumor lesion can be clearly visualized early at 30 min p.i. and exhibited tenacious radioactivity retention up to 2 h p.i., compared with that of muscle, thorax, liver, and other normal tissues. MicroPET imaging of mice bearing A375 human amelanotic melanoma and turpentine-induced inflammation revealed low radioactivity accumulation in tumor and inflammation lesions, which is consistent with those observed in biodistribution studies (Figure 5(a)). Quantitative analysis of tumor ROIs revealed a significantly different radioactivity accumulation between B16F0 tumor and A375 tumor at 2 h p.i. (7.03 ± 1.05 and 2.91 ± 0.41 %ID/g, resp., *P* < 0.05; Figure 5(b)). Owing to a rapid clearance of [^18^F]FPBZA from most of normal tissues, the tumor-to-muscle, and tumor-to-liver ratios increased with time and reached 6.53 ± 0.55 and 4.18 ± 0.35 at 2 h p.i., respectively. An appreciable radioactivity accumulation in bladder and feces was noticed at 30 min p.i., indicating that [^18^F]FPBZA was rapidly excreted through both renal and hepatobiliary routes.

**Figure 6:**
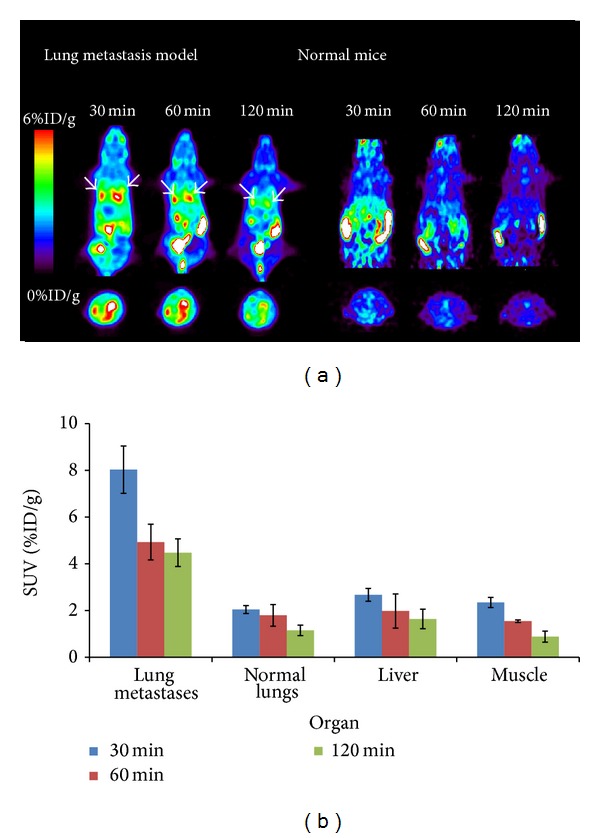
(a) Representative coronal (top) and transaxial (bottom) [^18^F]FPBZA microPET images of C57BL/6 mice bearing B16F0 melanoma lung metastases and normal mice. Static microPET images were obtained at 0.5, 1, and 2 h after bolus injection of 1.85–3.7 MBq of [^18^F]FPBZA via the tail vein. Arrows indicated tumors. (b) Quantification of the uptake of [^18^F]FPBZA by B16F0 melanoma lung metastases, normal lungs, liver, and muscle derived from microPET images after intravenous injection. SUVs were expressed in  %ID/g ± SD (*n* = 3).

[^18^F]FPBZA microPET scan clearly delineated the pulmonary metastases of B16F0 melanoma in C57BL/6 mice with high contrast (Figure 6(a)). The radioactivity accumulation in lungs with melanoma metastases (4.77 ± 0.36 %ID/g) was 4.1-fold higher than that in normal lungs (1.16 ± 0.23 %ID/g, *P* < 0.05) at 2 h p.i. (Figure 6(b)). High tumor-to-liver and tumor-to-muscle ratios were also noticed (3.29 ± 0.49 and 4.04 ± 0.34 %ID/g at 2 h p.i., resp.; Figure  S2). [^18^F]FPBZA microPET/CT scan of the mouse bearing pulmonary B16F0 metastases also displayed appreciable tumor uptake and gave high contrast images (Figure  S3). Pulmonary B16F0 metastases can be clearly seen and localized by the coregistration of microPET images with CT.

## 4. Discussion

Melanoma is an increasingly important public health problem around the world. Highly invasive melanoma is currently the fifth most frequently diagnosed cancer in men and the sixth in women in the United States [1]. The survival rate of melanoma has been steadily improved in decades, which can be greatly attributed to successful detection of melanoma in its early stage. Earlier diagnosis and treatment could promote survival rate and reduce healthcare expenditure. In this study, we sought to develop [^18^F]FPBZA as a specific PET probe for melanoma and presented a preclinical assessment of [^18^F]FPBZA in melanoma animal models.

DMSO, a highly polar and aprotic solvent, is very suitable for conducting nucleophilic substitution reaction. In our previous study, preparing 9-(4-[^18^F]-fluoro-3-hydroxymethylbutyl)guanine by radiofluorination of its tosyl precursor in DMSO gave higher radiochemical yield than that in acetonitrile [23]. In this study, conducting nucleophilic ^18^F-fluorination of compound** 2** with K[^18^F]/K2.2.2 in DMSO afforded noncarrier-added [^18^F]FPBZA with high radiochemical yield. An average radiolabeling efficiency of >90% was achieved within 5 min (*n* = 5). [^18^F]FPBZA preparation via one-step reaction was straightforward, convenient, and reliable, which was a significant advantage over [^18^F]FBZA.

A series of cellular uptake studies revealed a rapid and high uptake of [^18^F]FPBZA in high melanin-expressing B16F0 melanoma cells, reaching 2.58 ± 0.21 and 4.12 ± 0.20 %AD/10^6^ cells after 5 and 120 min of incubation, respectively. However, in A375 tumor cells, an amelanotic melanoma cell line with high sigma receptor expression (1.8 mmol/mg protein), only limited cellular uptake was observed (0.31 ± 0.06 %AD/10^6^ cells) after 2 h of incubation [24]. Accumulation of [^18^F]FPBZA in melanotic B16F0 cells was thirteenfold higher than that in amelanotic A375 cells. To confirm that the specific binding of [^18^F]FPBZA in B16F0 cells is attributed to cellular melanin, B16F0 melanoma cells were pretreated with various concentrations of NB-DNJ. NB-DNJ can effectively inhibit the cellular tyrosinase activity and results in an appreciable loss of pigmentation in the treated melanoma cells. The uptake of [^18^F]FPBZA in 1 mM NB-DNJ-pretreated B16F0 melanoma cells dramatically reduced to 0.34 ± 0.23 %AD/10^6^ cells after 2 h of incubation, similar to that in A375 amelanotic cells. Taken together, the results of in vitro studies clearly demonstrated that [^18^F]FPBZA was a melanin-specific agent and the accumulation of [^18^F]FPBZA in melanoma cells was correlated with the cellular melanin content.

The distribution of [^18^F]FPBZA in B16F0 melanoma-bearing mice displayed rapid and high tumor uptake and persisted up to 2 h p.i, comparable to those reported for [^18^F]FBZA and [^18^F]MEL050 (6.57 %ID/g and 9.4 %ID/g, resp.,) [17, 25]. Although the radioactivity in muscle and blood of [^18^F]FPBZA was higher than those of [^18^F]FBZA and [^18^F]MEL050, high tumor-to-muscle and tumor-to-blood ratios (6.60 and 3.52 at 2 h p.i., resp.,) suggested favorable pharmacokinetic profile for specific targeting of melanoma. [^18^F]FPBZA PET would be a sensitive modality for noninvasive imaging of melanoma in vivo. However, the slightly increasing bone radioactivity implied that defluorination of [^18^F]FPBZA in living subjects seems likely and may interfere the detection of bone metastasis of melanoma.

It has been reported that the density of sigma receptors are high in liver and kidneys [26], while the results of biodistribution study and microPET imaging did not show appreciable uptake of [^18^F]FPBZA in these organs. Moreover, high radioactivity accumulation in the pigmented eyes of C57BL/6 mice but not in the unpigmented eyes of BALB/c nude mice indicated the specific binding of [^18^F]FPBZA to cellular melanin in vivo. Taken together, the [^18^F]FPBZA accumulation in the B16F0 tumor xenograft was melanin-specific but not sigma receptors-mediated.

[^18^F]FDG is currently the most commonly used PET tracer for the detection and staging of cancers, and also for the evaluation of therapeutic efficacy in the clinic. In comparing the microPET images of the subcutaneous B16F0 tumor model, the tumor-to-muscle ratio for [^18^F]FPBZA at 2 h p.i. reached 6.53 ± 0.55, which was slightly higher than the tumor-to-background ratio of 5.8 ± 0.5 for [^18^F]FDG [27]. Furthermore, [^18^F]FDG is not a tumor-specific probe. The poor selectivity of [^18^F]FDG in distinguishing tumor from inflammation is the major cause of false-positive findings in clinical PET applications. In contrast to [^18^F]FDG, [^18^F]FPBZA showed no perceptible accumulation in the inflammatory lesion. [^18^F]FPBZA PET may be applied for specific detection of melanoma without interference from inflammation.

[^18^F]FPBZA PET was also employed to monitor metastatic lesions with an experimental pulmonary metastasis mouse model. [^18^F]FPBZA microPET scan displayed remarkable radioactivity accumulation in lungs of pulmonary metastases-bearing mice compared with those of normal mice. The tracer uptake in muscle and liver was also significantly lower than that in pulmonary tumor lesions, rendering favorable tumor-to-normal tissues ratios. However, significant [^18^F]FDG uptake in the heart and the interscapular brown fat could interfere with the delineation of pulmonary metastatic lesions [18, 28, 29]. The results of our study demonstrated that [^18^F]FPBZA was a melanin-specific PET probe for imaging melanoma, either a subcutaneous xenograft or pulmonary metastases. Liver was another common site that readily developed distant metastases from the cutaneous malignant melanoma (15–20%) [4]. In comparison with [^18^F]FBZA, the low liver uptake and favorable tumor-to-liver ratio of [^18^F]FPBZA observed in subcutaneous melanoma animal models (1.74 %ID/g versus 2.35% ID and 4.49 versus 2.80) suggested that it may be more suitable for imaging liver metastases of melanoma [17].

A possible explanation for high melanoma uptake of [^18^F]MEL050 was the positive charge in the nicotinic ring of [^18^F]MEL050. The binding of the cationic substances to the melanin can be reinforced by ionic interaction [30]. In contrast, the benzene ring of [^18^F]FBZA or [^18^F]FPBZA was electrically neutral. Thus, the further improvement of ^18^F-labeled benzamide-derivative radiotracers could focus on the optimization of the pharmacokinetic properties by introducing the cationic moiety.

Several ^18^F-labeled *α*-melanocyte-stimulating hormone (*α*-MSH) analogues were developed via the conjugation of [^18^F]SFB or 4-nitrophenyl 2-[^18^F]fluoropropionate and proven to be as excellent PET imaging agents for melanoma [31, 32]. However, the preparation of these ^18^F-labeled *α*-MSH analogues required multiple radiosynthetic steps and significantly longer reaction time and made them less attractive for clinical application [33].

## 5. Conclusion

[^18^F]FPBZA can be readily prepared with high radiochemical yield and radiochemical purity. This preparation method for [^18^F]FPBZA was very simple and straightforward, compared with [^18^F]FBZA. [^18^F]FPBZA showed highly specific uptake in the high melanin-expressing melanoma both in vitro and in vivo studies. Our results suggested that [^18^F]FPBZA may be a promising PET probe for the diagnosis of primary melanoma and the metastatic lesions.

## Supplementary Material

Supplementary material included analytic HPLC chromatogram of [^18^F]FPBZA after incubation 2 h incubation in serum (Figure S1), quantitative analysis of [^18^F]FPBZA microPET images in C57BL/6 mice bearing B16F0 melanoma lung metastasis (Figure S2) and representative microPET/CT images of C57BL/6 mouse bearing B16F0 melanoma lung metastases at 2 h postinjection of [^18^F]FPBZA (Figure S3).

## Figures and Tables

**Table 1:** Biodistribution of [^18^F]FPBZA in C57BL/6 mice bearing either B16F0 tumor or inflammation and Balb/c nude mice bearing A375 tumor after intravenous injection. Mice were injected with 3.7 MBq of [^18^F]FPBZA through lateral tail vein. Results were expressed as the percentage of injection dose per gram of organ (%ID/g) (*n* = 5; mean ± SD).

	B16F0 tumor			A375 tumor	Inflammation
	30 min	60 min	120 min	120 min	120 min
Blood	3.78 ± 0.41	2.91 ± 0.18	2.17 ± 0.10	2.48 ± 0.08	2.14 ± 0.22
Heart	3.30 ± 0.42	2.90 ± 0.23	2.26 ± 0.15	2.51 ± 0.21	2.30 ± 0.31
Lung	3.07 ± 0.42	2.37 ± 0.19	1.05 ± 0.32	1.57 ± 0.19	1.19 ± 0.06
Liver	3.43 ± 0.27	2.67 ± 0.25	1.74 ± 0.25	2.04 ± 0.27	1.78 ± 0.21
Stomach	3.17 ± 0.81	2.11 ± 0.58	1.28 ± 0.13	1.48 ± 0.45	1.31 ± 0.07
Si	3.98 ± 1.05	2.45 ± 0.38	1.32 ± 0.19	1.94 ± 0.09	1.48 ± 0.13
Li	4.38 ± 0.58	2.33 ± 0.50	2.41 ± 0.38	2.67 ± 0.19	2.13 ± 0.69
Spleen	3.13 ± 0.52	2.37 ± 0.49	1.42 ± 0.25	1.61 ± 0.58	1.76 ± 0.15
Pancreas	3.09 ± 0.67	2.09 ± 0.31	1.08 ± 0.07	1.07 ± 0.32	1.30 ± 0.26
Muscle	2.83 ± 0.46	1.87 ± 0.21	1.16 ± 0.09	1.26 ± 0.02	1.28 ± 0.07
Bone	2.68 ± 0.38	2.93 ± 0.40	3.21 ± 0.16	3.53 ± 0.10	3.48 ± 0.85
Tumor	10.37 ± 1.13	8.31 ± 1.00	7.81 ± 0.82	3.00 ± 0.71	
Kidney	4.92 ± 0.74	3.44 ± 0.44	2.00 ± 0.12	1.97 ± 0.59	2.27 ± 0.79
Eyeball	12.13 ± 0.94	9.74 ± 1.32	7.69 ± 1.56	2.91 ± 0.45	6.55 ± 0.45
Brain	2.24 ± 0.23	2.07 ± 0.15	1.49 ± 0.21	1.89 ± 0.37	1.64 ± 0.16
Inflammation					1.67 ± 0.56
Uptake ratio					
Tumor-To-Muscle	3.72 ± 0.53	4.47 ± 0.58	6.60 ± 1.00	2.72 ± 0.05	
Tumor-To-Liver	2.90 ± 0.23	3.12 ± 0.33	4.49 ± 0.60	1.70 ± 0.24	
Tumor-To-Blood	2.52 ± 0.07	2.86 ± 0.34	3.52 ± 0.51	1.38 ± 0.04	
Tumor-To-Brain	4.54 ± 0.20	4.03 ± 0.43	5.19 ± 0.93	1.72 ± 0.03	
Tumor-to-lung	3.43 ± 0.56	3.53 ± 0.51	9.01 ± 4.85	2.27 ± 0.32	
