# Supplementary material for: Synthesis and Preclinical Characterization of [18F]FPBZA: A Novel PET Probe for Melanoma
Source: Biomed Res Int. 2014 Sep 1;2014:912498. doi: 10.1155/2014/912498 (PMC4165567; doi:10.1155/2014/912498)
Supplement: Supplementary file 1 — Supplementary material included analytic HPLC chromatogram of [18F]FPBZA after incubation 2 h incubation in serum (Figure S1), quantitative analysis of [18F]FPBZA microPET images in C57BL/6 mice bearing B16F0 melanoma lung metastasis (Figure S2) and representative microPET/CT images of C57BL/6 mouse bearing B16F0 melanoma lung metastases at 2 h postinjection of [18F]FPBZA (Figure S3). [file 912498.f1.docx]

Supporting information

Synthesis and preclinical characterization of [^18^F]FPBZA, a novel PET probe for melanoma

Shih-Yen Wu^a^, Shih-Pin Huang^a^, Yen-Chen Lo^a^, Ren-Shen Liu^b^, Shyh-Jen Wang^b^, Wuu-Jyh Lin^c^, Chih-Chieh Shen^d,^*, Hsin-Ell Wang^a,^*

^a^Department of Biomedical imaging and Radiological Sciences, National Yang-Ming University, Taipei, Taiwan

^b^Department of Nuclear Medicine, Faculty of Medicine, National Yang-Ming University, Taipei, Taiwan

^c^Institute of Nuclear Energy Research, Atomic Energy Council, Taoyuan, Taiwan

^d^Department of Nuclear Medicine, Cheng-Hsin General Hospital, Taipei, Taiwan

*Corresponding authors:

Hsin-Ell Wang, Ph.D.

Tel: 886-2-28267215; Fax: 886-2-28201095

E-mail: hewang@ym.edu.tw

Mailing address: Department of Biomedical imaging and Radiological Sciences, National Yang-Ming University, No. 155, Li-Nong St., Sec. 2, Pei-tou, Taipei 11221, Taiwan

Chih-Chieh Shen, M.D.

Tel: 886-2-28264400 ext 5713; Fax: 886-2-28267472

E-mail: chihchieh0330@yahoo.com.tw

Mailing address: Department of Nuclear medicine, Cheng-Hsin General Hospital, No.45, Cheng Hsin St., Pai-Tou,Taipe 11220, Taiwan

Figure S1: Analytic HPLC chromatogram of [^18^F]FPBZA after incubation 2 h incubation in serum.

*MicroPET/CT imaging*

MicroPET/CT imaging was performed at 2 h after intravenous injection of [^18^F]FPBZA (18.5 MBq) (FLEX Triumph Regular FLEX X-OCT, SPECT CZT 3 Head System, LabPET4 Tri-modality system, GE Healthcare, Northridge, CA). The mouse were anesthetized by inhalation of 2% isoflurane in 2 L/min oxygen in the prone position. CT imaging was conducted following the acquisition of microPET images (X-ray source: 50 kVp, 0.28 mA; 512 projections). The co-registration of microPET and CT images was performed using VIVID (Volumetric Image Visualization, Identification and Display) software (based on Amira 4.1 platform). The microPET images were reconstructed to produce an image volume of 240×240×31 with an image resolution of 1.0 mm. The CT images were also reconstructed using the Feldkamp cone-beam algorithm for filtered backprojection in an image volume of 512×512×512 with an image resolution of 0.1 mm. The CT data were not corrected for scatter or beam hardening. VIVID software was also used for the image fusion of microPET images and microCT images. After registration, the image of microPET/CT had 256×256×256 voxels in an isotropic 0.24 mm voxel size.

Figure S2: The tumor-to-normal organ ratios of [^18^F]FPBZA in C57BL/6 mice bearing B16F0 melanoma lung metastasis-bearing C57/BL6 mice. All data are expressed as mean ± SD (n = 3 per group).


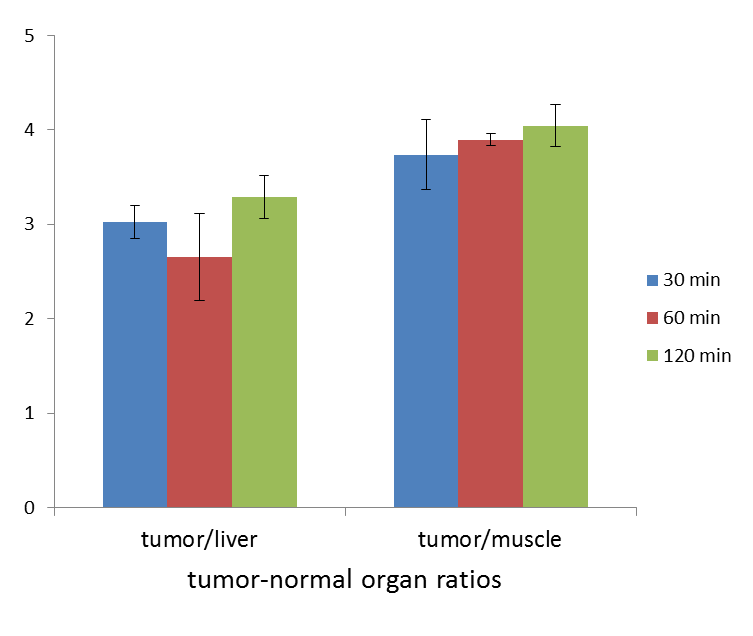


Figure S3: Representative CT images, microPET images and microPET/CT fusion images of C57BL/6 mouse bearing B16F0 melanoma lung metastases at 2 h after bolus injection of 18.5 MBq of [^18^F]FPBZA into the tail vein.


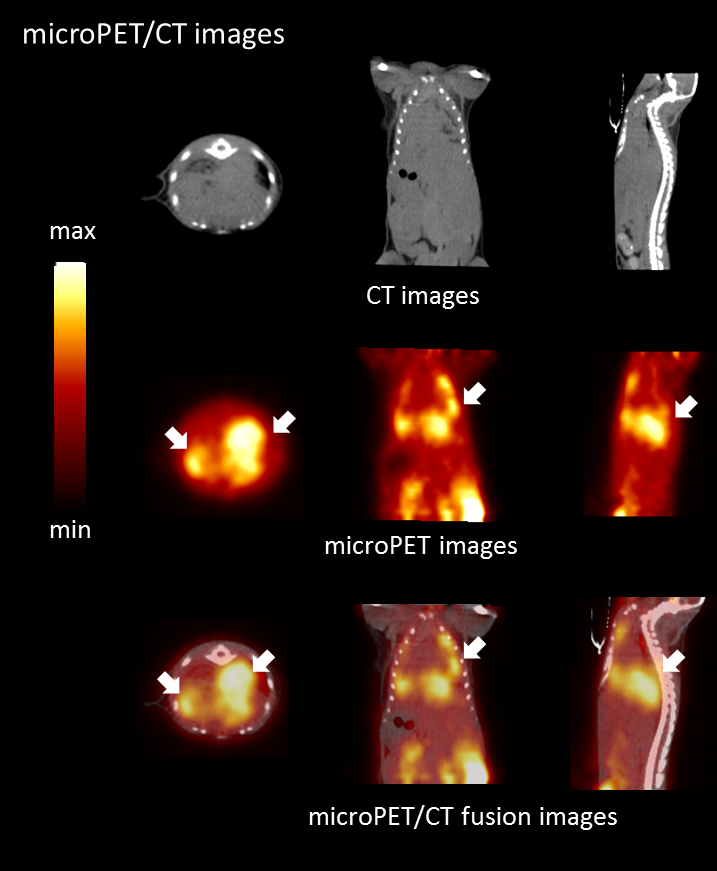


Figure S3: Analytic HPLC chromatogram of [^18^F]FPBZA after incubation 2 h incubation in serum.
